# Full-profile search–match by the Rietveld method

**DOI:** 10.1107/S160057671900342X

**Published:** 2019-05-14

**Authors:** Luca Lutterotti, Henry Pillière, Christophe Fontugne, Philippe Boullay, Daniel Chateigner

**Affiliations:** a Dipartimento Ingegneria Industriale, Università di Trento, Italy; b Thermo Fisher Scientific, INEL SAS, Artenay, France; c CRISMAT – ENSICAEN, Université de Caen Normandie, Normandie Université, France

**Keywords:** Rietveld method, search–match, diffraction, full profile, quantitative analysis

## Abstract

A new search–match procedure has been developed and tested which, in contrast to previously existing methods, does not use a set of lines identified from a diffraction pattern, but an optimized Rietveld fitting on the raw data. Modern computers with multicore processors allow the routine to be fast enough to perform the entire search in a reasonable time using quite large databases of crystal structures. The search–match is done using the crystal structures for all phases and the instrumental geometry, and as such can be applied to every kind of diffraction experiment, including X-rays, thermal/time-of-flight neutrons and electrons. The methodology can also be applied to nanocrystalline samples for which peak identification may be a problem. A web interface has been developed to permit easy testing and evaluation of the procedure. The quality of the results mainly depends on the availability of the sought phase in the structure database. The method permits not only phase identification but also a rapid quantification of the phases and their gross microstructural features, provided the instrumental function is known.

## Abstract 2

A new fully automatic search–match procedure based on a Rietveld full-pattern-fitting method is presented. The method does not rely on peak positions and permits not only phase identification but also a quantification of the phases and their gross microstructural features.

## 1. Introduction   

The history of search–match in the crystallographic context coincides with the history of Hanawalt’s work and the International Center for Diffraction Data (ICDD), and a good account of those early days is given in the paper by Hanawalt (1986 ▸).

Following the ‘Hanawalt’ manual search, the appearance of the personal computer revolutionized search–match, making it faster, more powerful and easier to use (Smith & Gorter, 1991 ▸; Langford & Louër, 1996 ▸). More lines could be used, subsets and chemical information helped the identification of the correct phases, and errors were taken into account. Most of the subsequent work has been devoted to correctly determining peak positions in an automated way and using, as much as possible, the intensity information. These search–match methods are conducted in two steps: the first step is a classical search–match using the identified lines, and then, in a second step, the possible structures are compared directly as lines with the powder pattern to further refine the choice manually.

The majority of search–match improvements have resulted from the algorithms and software being able to provide increasingly reliable and automated results, limiting human intervention. In parallel, another major effort has been dedicated to increasing the number of ‘cards’ from Hanawalt’s days, using experimental data from different laboratories and collected by ICDD into the Powder Diffraction File (PDF) databases. In the past ten years, large improvements in both the number and the reliability of cards have been obtained by including calculated ‘cards’ or patterns. Crystal structures from different databases, notably the Inorganic Crystal Structure Database (ICSD; Hellenbrandt, 2004 ▸) and the Crystallography Open Database (COD; Gražulis *et al.*, 2009 ▸, 2012 ▸), have been added to search–match procedures. As these databases provide crystal structures for identified phases, one can proceed with phase quantification using the Rietveld method (Rietveld, 1967 ▸, 1969 ▸), which does not require any standard or knowledge of reference intensity ratio factors.

In 2002, Le Meins *et al.* (2003 ▸) organized a search–match round robin, and the overall result was satisfactory. It proved that ‘a good search–match software, in expert hands, combined with an up-to-date PDF can perform efficiently in solid state phase identification’. But it also showed that only one participant was able to succeed in identifying all compounds, and ‘in expert hands’ can still be interpreted as being far from an automatic procedure for beginners. Furthermore, the investigation of nanostructured compounds, or in general not perfectly crystallized samples, was not a concern in this round robin. For such compounds, the increased peak broadening makes reliable peak finding and indexing more difficult to perform, and unfortunately increased instrument resolutions are of no help. Another general problem is that the traditional search–match, in certain cases, identifies a large number of phases, which makes it difficult for the non-expert to discern which ones are really present and which are not. This is in part due to the ranking mechanism, which uses mainly a list of identified peaks with the main emphasis on number and position as the true overlapped intensity cannot be exploited.

As a general trend, actual search–match procedures are very efficient in identifying phases in compounds with well defined and crystallized structures, coming from synthesis or chemical laboratories. But this efficiency decreases for less well defined structures, because of their poorer crystallinity or/and their strong inter- and intra-phase overlap, *e.g.* in soil samples or raw industrial materials.

In 2009, a project named ‘Nanoair’ was founded by the European Community to develop a fully automatic and remotely controlled instrument to collect nano-powders from the air and perform a complete analysis for particle dimensions and composition (phase composition by diffraction). The analysis part of the project focused on performing an automatic phase identification and quantification using the Rietveld method (Werner *et al.*, 1979 ▸; Hill & Howard, 1987 ▸; Bish & Howard, 1988 ▸). The results of the phase identification can be used in a further step for the Rietveld-based quantification only when the crystal structures of identified phases are known. Therefore, we decided to work on a new procedure to merge the phase quantification and search–match in a single step using the Rietveld method. In that project, the software/procedure FPSM (full-profile search–match) has been developed. Since then, the method and software have been tested and improved, and the full theoretical basis, details of the implementation and some test results of the search–match are presented in the following. Structural and profile parameters are not fully refined in the Rietveld quantification in order to conduct the procedure automatically. When more accurate quantification is required, we advise the use of a full Rietveld refinement employing the identified phases or application of another of the available quantification methods, in particular if unidentified phases are present (Scarlett & Madsen, 2006 ▸; Toraya, 2016 ▸, 2018 ▸). We will not discuss the quantification here, only the search–match.

## 2. Theory   

### 2.1. Automatic Rietveld refinement   

The entire search and profile fitting algorithm is based on the Rietveld method (Rietveld, 1969 ▸). In particular we use a special implementation, as in the *MAUD* software (Lutterotti, 2010 ▸), that differs from the classical Rietveld implementations in two main points:

(1) The peak profile function is composed of two different contributions: the instrument and sample broadening. The two contributions are directly convolved into the resulting peak broadening, and in the analysis we do not refine the instrument broadening contribution, only the crystallite sizes and r.m.s. microstrains.

(2) The refinement strategy, *i.e.* how the parameters are refined in successive order, is automatically decided by the algorithm and does not require human intervention or decision.

Only the second point is strictly necessary for the Rietveld search–match algorithm, but the first is convenient as it directly provides the quantities of interest.

The general formula used for the Rietveld computation is the following:
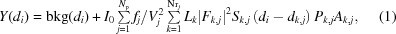
where bkg() is the background function, *I*_0_ the diffraction part of the incident intensity, *N*_p_ the number of phases, *f*_*j*_ the volume fraction of phase *j*, *V*_*j*_ its unit-cell volume, Nr_*j*_ the number of reflections of phase *j* inside the pattern range considered, *L*_*k*_ the Lorentz–polarization factor for reflection *k*, *F*_*k*,*j*_ its structure factor, *S*_*k*,*j*_() the peak shape function of reflection *k* of phase *j*, *d*_*k*,*j*_ its position in *d*-space coordinates, *P*_*k*,*j*_ the texture or preferred orientation factor, and finally *A*_*k*,*j*_ the absorption factor. We prefer the variable *d*_*i*_ as coordinate for the pattern instead of 2θ because we use it here for both angular dispersive and energy dispersive [*e.g.* neutron time-of-flight (TOF)] techniques.

Now we detail some of the functions and models used in the Rietveld algorithm implementation.

For the background function we use a simple polynomial function plus an additional Gaussian function at zero 2θ (but not for TOF/energy dispersive patterns) to represent the decay that we can find in some experiments close to the incident beam. In the case of electron diffraction in transmission electron microscopy (TEM), such a strong contribution is always present, resulting in a strong increase of the background at large *d* values.

For the peak profile we use an asymmetrical pseudo-Voigt function as described elsewhere (Enzo *et al.*, 1988 ▸; Lutterotti & Scardi, 1990 ▸). The instrumental broadening part can be determined in advance using a standard sample (Scardi *et al.*, 1994 ▸), and to simplify its input it was made compatible with that in the *MAUD* software (in *MAUD* we added a menu function to export a CIF containing the data and the instrument characteristics and broadening ready to be loaded and used in the web interface of the FPSM program). If the experimental details are not supplied alongside the data with a suitable CIF, the web interface can be used to specify some general categories for instrumental broadening to represent usual cases and any additional information needed regarding the experimental conditions. An imperfect definition of the instrumental contribution does not prevent the search–match from working correctly (unless a low-resolution instrument broadening is used for a high-resolution instrument and well crystallized sample), but it will give wrong results for the crystallite size and microstrain values and is likely to make the search more difficult.

The broadening due to the crystallite size and microstrain for each phase is computed using a simple inversion of the model described by de Keijser and co-workers (de Keijser *et al.*, 1983 ▸; Delhez *et al.*, 1983 ▸). In the Rietveld algorithm we first compute the integral breadth (both the Gaussian β_g_ and Cauchy β_c_ parts) for the pseudo-Voigt function from the value of crystallite size 〈*D*_V_〉 and r.m.s. microstrain 〈ε〉^1/2^ according to
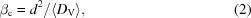


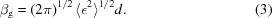



The integral breadth is then convoluted analytically with the pseudo-Voigt function, representing the symmetric instrument broadening part, and further numerically convoluted with the asymmetry function as described by Lutterotti & Scardi (1990 ▸).

The Lorentz–polarization correction is computed according to four different geometries: Bragg–Brentano, Debye–Scherrer, transmission (TEM or X-ray diffraction) and neutron TOF. No correction for monochromator or highly polarized beams like on a synchrotron is used, as in nearly all cases it will not greatly affect the results. In general for a better refinement, after the phase identification and first quantification using this method, a more accurate complete Rietveld refinement may be performed using all corrections and required models.

We decided not to apply a texture correction in the present FPSM, for three reasons:

(1) A preferred orientation correction such as the March–Dollase (Dollase, 1986 ▸) requires human intervention for determining the preferred orientation direction.

(2) The spherical harmonic model (Popa, 1992 ▸) is time consuming and it is not advisable to use it in general for phase quantification as it may not correctly represent the effective sample texture (Lutterotti, 2012 ▸).

(3) The use of a true orientation distribution function requires additional experiments (Ferrari & Lutterotti, 1994 ▸; Wenk *et al.*, 1994 ▸; Lutterotti *et al.*, 1997 ▸) to cover the orientation space.

The approximation of a randomly oriented sample may cause problems for the identification of certain clay minerals, and at present we are working on a specific solution for this case.

For the absorption-volume correction, the formulation depends on the diffraction geometry and four models were implemented: (1) Bragg–Brentano with θ/2θ measurements, for which the absorption factor is a constant and can be ignored for bulk samples, (2) flat sample in reflection with 2θ measurement only, where the absorption factor is calculated as a function of 2θ from the incident beam angle and phase composition, (3) Debye–Scherrer camera with the absorption-volume correction for a cylinder, and (4) no absorption correction for X-ray diffraction or TEM transmission and TOF neutrons.

Finally, we explain how an automatic Rietveld refinement is performed in the code each time we evaluate the presence of a phase. The execution strategy is very similar to a Rietveld analysis focused on phase quantification and not on crystal structure refinement. We remind the reader that the goal is to obtain a quick correct analysis and avoid as much as possible a divergent solution. Thus we reduce the number of refined parameters to the minimum possible and emphasize physically meaningful parameters/formulation even if this does not result in the best possible fit of the pattern. The refinement strategy is reduced to three steps, which correspond to the first three steps performed by a Rietveld expert when performing a pattern fitting. The first step consists of refining only the background and scale parameters (each *f*_*j*_ *I*_0_ as phase scale parameters), the second step additionally refines peak positions, and finally in the third step the peak profile parameters are introduced. No crystal structure is refined.

To go into more details on our specific Rietveld formulation, for each step we perform the least possible number of iterations to reduce the execution time. We actually use two different types of refinements. One is used during the search or scan through all the selected structures. It is focused on speed and it does not use more than three iterations per step. The other is used for the phase quantification and it requires a better fitting. It is conducted only at the end of the selection of a new phase and thus it does not need to be as fast as possible. During the first step, three to five background parameters are refined in the polynomial function (depending on how large the data range is): the phase scale parameters and a decay function for the diffuse scattering halo around zero. This step is also used to get a first estimate of all volume fractions. As the volume fractions should amount to a total of 100%, at the end of each iteration, the volume fractions are normalized and the normalization factor is used to change the incident intensity *I*_0_ [see equation (1)]. We keep this particular procedure to remain compatible with the calculations in *MAUD*, in which working with volume fractions (not simply phase scale factors) is necessary when dealing with multiple data sets (from different instruments) or layered and multilayer samples.

After the first step, if there are multiple phases and one or more of them results in a volume fraction below a minimum threshold (*f*_*R*0_, normally between 0.5 and 0.1%), these are eliminated from the refinement. For the remaining phases, in the second step we add the unit-cell parameters to the refinement, but only for the phases having a volume fraction over a given threshold (*f*_*R*1_, around 5–10%). In addition, a classical 2θ or constant *d*-space shift is refined to account for goniometer misalignment or measurement errors. Regarding the cell parameters, it is not advisable in our case to refine all of them, especially for triclinic or monoclinic phases, so during the search algorithm we use instead a volume expansion or contraction parameter, thereby reducing the total number of refined parameters added in this cycle to (1 + *N*_p_). In the third step we add the crystallite size and microstrain parameters to the refinement, for each phase with a volume fraction over a specific threshold (*f*_*R*2_). During this step we refine also an overall isotropic Debye–Waller factor common to all atoms of all phases.

### 2.2. Search and match algorithm structure   

**Figure 1:**
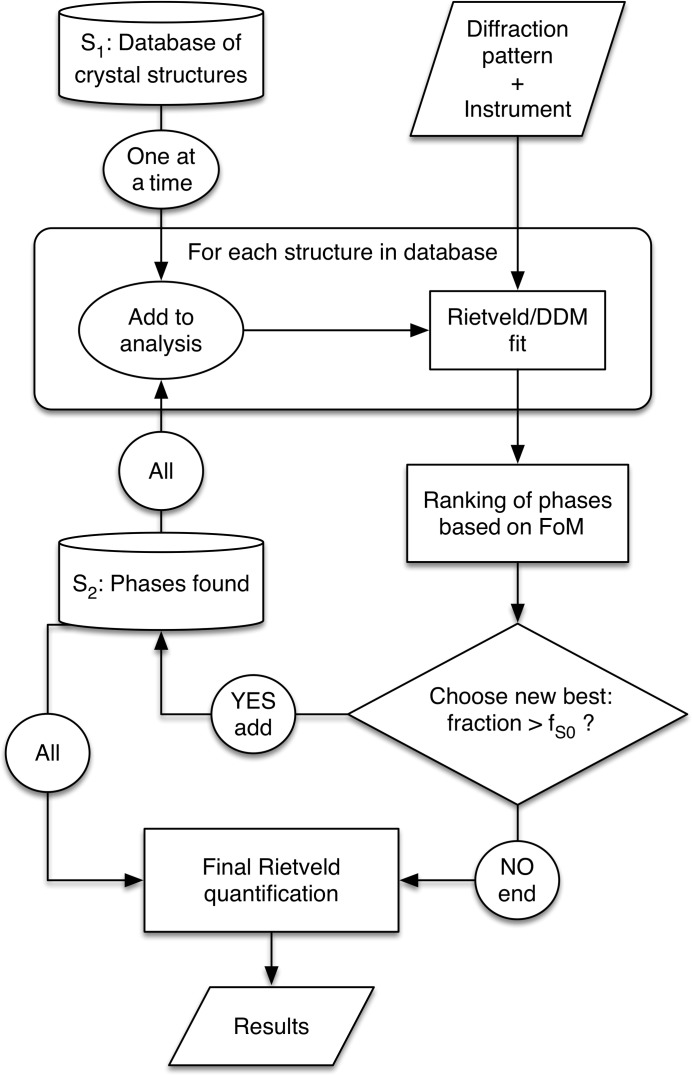
Schematic layout of the FPSM algorithm.

We describe in this section the main algorithm used for what we call the full-profile search–match (Fig. 1 ▸). We start from a crystal structure database restricted to match the elemental composition conditions. In principle the method may work with all the possible phases without any composition restriction, but we have to consider the computing time, and limiting the number of phases using some restrictions on the composition or type of sample (*e.g.* mineral, inorganic…) means that a search can be performed in a few seconds or minutes.

Let us call *S*_1_ the restricted crystal structures subset, composed of *N* potential phases, and *S*_2_ the subset containing the identified phases, initially empty. Each phase of *S*_1_ is then evaluated against the experimental pattern by performing an automatic Rietveld refinement as described at the end of the previous section (three-step procedure). At the end of this run, the *N* phases are ranked on the basis of a figure of merit (FoM, defined later) and the rank-1 phase is moved to *S*_2_. All phases of *S*_2_ (at the moment just one) are then added in turn to each of the (*N* − 1) remaining phases of *S*_1_ for another automatic Rietveld run over these (*N* − 1) combinations of phases. The combination (*S*_2_ + 1 phase from *S*_1_) providing the best FoM indicates the next phase that has to be moved to *S*_2_, leaving in *S*_1_ only (*N* − 2) phases. At this point a more accurate Rietveld refinement is performed to evaluate the volume fractions for all the phases found up to now. If one of the *S*_2_ phases falls below a critical threshold (*f*_*S*0_, between 0.5 and 0.1%), the phase is moved out of *S*_2_ and another cycle starts to evaluate the next phase to add to *S*_2_. To avoid similar or duplicated phases in the analysis, a special check is done by comparing the newly found phase with each one in *S*_2_, and in the case of a positive match, the phase is not added but is removed also from *S*_1_ and the next phase in the rank is chosen. The check is done by fitting the calculated pattern of the last phase added to *S*_2_ with the other structures in turn. If the *R*_wp_ factor from the fitting is below a certain threshold, the two phases are considered similar. The process is carried out until either the phase removed from *S*_2_ as a result of being below the threshold *f*_*S*0_ is also the last one added (this means that no phases over that threshold were found in the last cycle) or there are no more phases left in *S*_1_ or the number of phases in *S*_2_ is over an imposed limit. We keep the last possibility as a precaution to stop the algorithm in case of a completely failed identification, giving rise to several phase additions without a real fit improvement. In our still brief experience with the algorithm, the latter happens when (i) severe errors are present in the pattern (including wrong data formats *etc*.); (ii) the pattern does not correspond to the declared experimental conditions; or (iii) neither the phase nor a closely related structure is present in the database.

By default, after each cycle, we eliminate from *S*_1_ not only the phases moved to *S*_2_ but also the ones resulting in a volume fraction below a threshold *f*_*S*1_, to speed up the next Rietveld ranking by having fewer structures in *S*_1_.

### 2.3. Figure of merit   

The simplest figure of merit that can be used to rank the crystal structures during one cycle is based on the *R*_wp_ factor (Prince, 1983 ▸), computable directly by the Rietveld fitting. As we are using it to test the suitability of a phase to reproduce a pattern and we aim to decide whether the phase is present or not, it may be reasonable to use the most general and best overall Rietveld index.

In such a case the figure of merit becomes
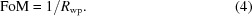
This FoM was also the first one used during testing and tuning of the algorithm. But we found it not completely suitable for seeking and sorting out the structures that are present in a pattern for several reasons, of which the main ones are as follows:

(1) The *R*_wp_ criterion does not distinguish between two similar phases from the database mainly differing by their cell parameter values. This may happen for two structures originally determined at different temperatures or with compositional modifications, for which the structure factors and unit-cell parameters do not change too much.

(2) *R*_wp_ privileges strong scattering phases. Phases with larger phase fraction should have greater weight.

(3) Phases with substantial broadening can significantly reduce *R*_wp_ by simply improving the background fitting.

To overcome these problems we propose a modified FoM, where the phase-related parameters are the ones of the phase for which we are evaluating the FoM:
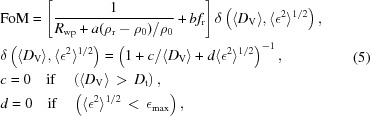
where ρ_0_ is the starting density, ρ_r_ is the refined density and *f*_r_ is the volume fraction of the structure for which we are evaluating the FoM from the Rietveld fit. The quantities *a*, *b*, *c* and *d* are weighting coefficients to be tuned for optimal performance. δ is a strong penalty function that reduces the FoM if the mean crystallite size of the phase is below a customizable threshold value (<*D*_t_, default is 20 Å) and/or the r.m.s. microstrain is too large (*e.g.* >∊_max_, default is 0.02). The term weighted by the coefficient *a* gives more importance to structures with the closest cell parameters to the observed ones. The term weighted by *b* favours the low scattering phases, so these are not excluded in virtue of the non-negligible volume fraction they are representing.

A correct tuning of the FoM parameters is important for the success of the algorithm, as the *R*_wp_ criterion alone is not sufficient to cover all the possibilities. But there is one additional note: the FoM as described here is useful only to compare and rank the crystal structures in one cycle, but not to follow the entire search–match progress or compare different cycles. This because each cycle, to identify the next phase, is different from the previous cycles (*S*_1_ and *S*_2_ are different) and the new best matching structure may have a higher penalty function. To follow the overall progress, *R*_wp_ or the WSS (weighted sum of squares; Toby, 2006 ▸) are better suited, and in general we always observe them decreasing quite a lot during the initial identifications and then levelling to a minimum when arriving at the minor phases.

## 3. Implementation   

The adoption of the FPSM procedure as a routine tool for quick automatic finding of more likely structures depends heavily on the speed of execution of the code. For this reason we tested in the implementation different possibilities of how to perform the calculation and how to store and parse the crystal structure database. For the implementation and testing we used both a portable computer with an i7 quad-core 2.8 GHz processor and a workstation with 2 × 2.93 GHz six-core Xeon processors, resulting in 158 gigaflops. Both systems were equipped with a solid state drive (SSD). The programming language used for the main program was C++, and we employed the database management system MySQL to store/retrieve the crystal structures. All the crystallographic computations (space-group interpretation, symmetry operations, reflection list and structure factor calculation) were carried out using the *Computational Crystallography Toolbox* (*cctbx*; Grosse-Kunstleve & Adams, 2002 ▸; Grosse-Kunstleve *et al.*, 2002 ▸). The latter proved very efficient in all the calculations and we used only the C++ implementation without the Python bindings. We added the scattering factors for electron diffraction to the *cctbx* library as tabulated by Peng *et al.* (1996 ▸), to enable a simple kinematical calculation of structure factors.

We tested two ways of preparing intensities of individual reflections:

(1) Preliminarily calculate the intensities and store them in the database.

(2) Calculate the intensities when the structures are loaded from the database.

The full computation took more time for the former than the latter. It was not shortened even if a quick SSD was used or the database was further optimized in the former. Thus the latter was adopted in the present computation. Moreover, the latter reduces the size of the database for parsing and gives flexibility in changing the radiation type. For the nonlinear least-squares algorithm we use a Marquardt algorithm (Marquardt, 1963 ▸), whose implementation was derived from the *MAUD* software (Lutterotti, 2010 ▸). Since numerical models were partly used in the formulation, all derivatives were computed numerically.

We discovered that finding minor phases with this method required a good reproduction of the background, as just the differences between experimental and calculated data points were used. The derivative differences method (DDM; Solovyov, 2004 ▸), on the other hand, does not rely on a good fit of the background as it minimizes first and second derivatives instead. So in FPSM, we implemented, in addition to the classical Rietveld method, the refinement indices and weighting schemes of DDM as reported in the aforementioned paper and manual (https://sites.google.com/site/ddmsuite/). In the search and match one can choose the Rietveld scheme, the DDM, or both Rietveld and DDM with a weight between the two.

In this first implementation and testing, all the structures imported into our database were extracted from the COD (Gražulis *et al.*, 2009 ▸, 2012 ▸) in CIF format (Hall *et al.*, 1991 ▸). In principle, we can use any other source providing crystallographic structures in CIF format, but for testing and our first database we used the COD because it contains the American Mineralogist Crystal Structure Database (Downs & Hall-Wallace, 2003 ▸) and the zeolites database (Baerlocher *et al.*, 2007 ▸) and it is the largest one comprising inorganics, organics and organometallics all together for which CIFs are freely available.

A dedicated interface has been written to perform the following tasks:

(1) Input all the CIFs from a local repository (at present, we use a clone of the COD, and we plan to implement later a function for updating the database when structures are added to the COD).

(2) Save the database in MySQL format and carry out all the maintenance or editing required.

(3) Check each structure for consistency: cell content with respect to chemical formula, space group and symmetry operations. At the present state of implementation we do not make any check for bond distances.

(4) Classify the phases as inorganic, organic or, when no clear distinction can be made from the raw chemical formula, unknown (organometallic mostly).

In the latest version of the database imported from the COD, 379 097 structures were loaded, 148 were removed because of retraction (Harrison *et al.*, 2010 ▸), 1567 were duplicates of others, and 1166 were not loaded because they contain an unrecognized space group, unrecognized atoms or chemical composition problems. Most of the unloaded structures were modulated structures (unrecognized space group) that are not supported in the present implementation. A few crystal structures were rejected because there was a significant mismatch between the chemical composition and the cell content; some were missing the atoms completely. For these files, even a check with the original article was not sufficient to solve the problem clearly (most of the time the CIF is a partial solution or a general framework with the atom-site content not specified; *e.g.* atom *X*, where *X* is one of the transition elements), and we prefer not to use them in our method. However, we could successfully identify, validate and use more than 99.2% of the structures from the COD.

The core of FPSM is devoted to the full-profile search–match only, and the implementation was based on the layout shown in Fig. 1 ▸. We chose to keep it independent of any graphical user interface for ease of compilation and to allow it to run on multiple platforms. This core part can be compiled and used as a library by any graphical interface and as such was included in the previously described program interface to build the database and test the algorithm. But to permit more widespread use, we have built an interface accessible through a web page.

**Figure 2:**
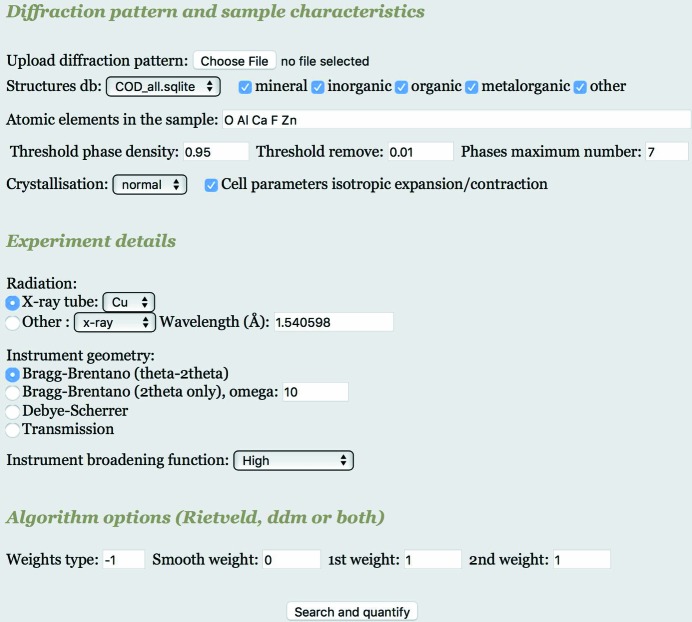
Web interface to test the algorithm using the COD database of crystal structures, as available at http://fpsm.radiographema.com. The user uploads a pattern in a double-column format, selects the instrument type and experimental conditions, restricts the search using composition and/or COD subsets, and finally runs the analysis. All the analyses reported in this paper were done using this web interface.

The web interface is shown in Fig. 2 ▸. It has a minimal input to keep it simple and is focused mainly on usability. It has three sections for the input. In the first section the user loads the pattern to be analysed as a simple text format with two columns: 2θ (or *d* spacing in the case of TOF measurements) and intensity. After loading the pattern the user may restrict the database to some subsets (*e.g.* mineral only or organic only) and specify a possible composition, and if a list of chemical elements is entered the program will exclude all the phases having at least one atom not in the list. This may be helpful to restrict the number of phases to search from and it is strongly advised to use it. In case of doubt many additional atoms may be included to lower the restrictions. Then three thresholds can be set by the web interface: the density threshold, the minimum amount of volume fraction (*f*_*S*1_) and the maximum number of phases. It is possible to specify if the sample contains mainly highly crystallized phases (‘Crystallization: high’), normal crystallization or nanocrystallized phases. This option will change the initial value used for the crystallites and tune the FoM differently to favour the type of phases selected. A final option is to refine only a volume expansion/contraction instead or to refine all cell parameters when the box is unchecked.

In the second section the user may specify the instrument geometry and details used in the measurement. Three different radiations are available: X-ray, neutron and electron. For X-rays, the user can select one of the conventional tubes with both *K*α_1_ and *K*α_2_; otherwise a strictly monochromatic radiation can be selected and the wavelength introduced. Three different geometries are available: Bragg–Brentano (flat sample reflection geometry), Debye–Scherrer and transmission. For Bragg–Brentano both possibilities of measurement, with θ/2θ or only 2θ, are available. Finally, we can specify the instrumental broadening, from very low as in a high-resolution synchrotron beamline to very broad as in TEM. The possible choices are just broad categories as the main purpose of the program is to perform the search–match (and rapid quantification) and not an accurate line broadening analysis. If the instrument broadening specified is sufficiently close to the real one, the program will give also a good estimation of the crystallization of the compound. For nanocrystallized samples, as the broadening is much greater than the instrument broadening (unless TEM is used), an error on the instrument broadening choice will not cause a large error in the size–strain parameters at the end of the analysis.

In the latest version of the program *MAUD* (available as a free download at http://maud.radiographema.com), we added the possibility to export a specific datafile in CIF format containing all the instrumental information needed by FPSM. When such a file is loaded as a pattern via the web interface, FPSM will ignore the web input from the ‘Experiment details’ section and the instrumental characteristics and broadening from the CIF will be used instead.

In the third section the algorithm options are available: the user can choose either only the Rietveld or a mixed DDM–Rietveld refinement at each step. This is specified with some weights for each type of refinement. The Rietveld refinement has a fixed weight of 1. Using the same weight of 1 for both the first and second derivative (DDM) ensures that the Rietveld, the first-derivative and the second-derivative methods all have the same importance in the algorithm. Entering 0 in both the first- and second-derivative fields corresponds to a Rietveld-only refinement, and using some large values instead produces mostly a DDM-like refinement. The ‘Weight type’ option is the same as that described in the DDM manual (Solovyov, 2004 ▸; https://sites.google.com/site/ddmsuite/). The ‘smooth fit’ corresponds to a zero-derivative DDM-like refinement or practically fitting the smoothed pattern with a Rietveld method. Its default value is zero, as at the moment we have not found any advantage to using it.

**Figure 3:**
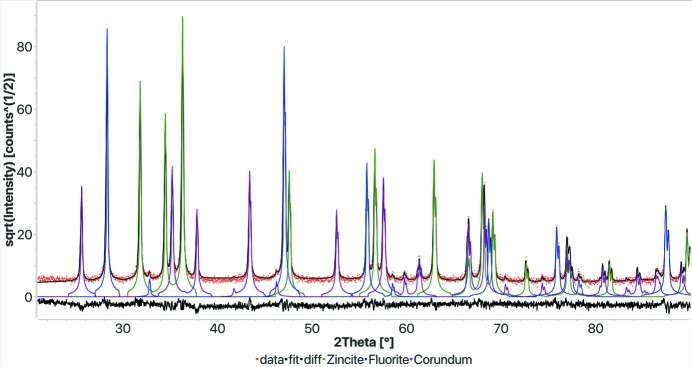
Results obtained after less than 10 s using CPD datafile 1*h*, with the composition shown in Fig. 2 ▸. The 1*h* diffraction datafile is from the quantitative phase analysis round robin (Madsen *et al.*, 2001 ▸). The figure and the table results have been visually reformatted for this article with respect to the original ones given in the output by the web interface. The same applies for all the subsequent figures.

When the input is finished in the three sections, pressing the ‘Search and quantify’ button will start the computation by submitting the job to a remote server. The server will select from the database the structures to be used and launch the FPSM algorithm. Another web page will appear (as will be shown later in Fig. 3 and Table 1), presenting a table giving the phases found, their percentage, and their crystallite sizes and microstrains, together with a graph illustrating the fitting result for visual evaluation.

## 4. Results and discussion   

Testing was done initially using the data set 1*h* from the round robin on quantitative analysis (Madsen *et al.*, 2001 ▸), which is available from the IUCr Commission on Powder Diffraction (CPD) web site (https://www.iucr.org/resources/commissions/powder-diffraction/projects/qarr/data). This is a simple analysis with three phases (corundum, fluorite, zincite) that was useful also for testing the accuracy of phase quantification. The tests focused on the final result and the speed of execution. In the following we will report the results using the web interface when imposing some compositional restrictions and the standalone program without restriction. This is because the web interface does not return a result when the computation becomes too long and the standalone program must be used instead. For this sample the analysis correctly identified all three phases both using no composition restriction and with restriction. The only difference between the two was the time taken to complete the analysis. On the workstation the computation time was 565 s without compositional restrictions but using the inorganic and minerals subsets of the COD, with a total of 174 064 phases. When restricting the composition to only phases containing the following atoms – Al, Ca, F, Zn, O, Mg, Na, Si, Cl – the computation time dropped to 19 s. If we specify only the atoms really present in the sample (Al, Ca, Zn, O, F) the total time falls below 10 s, most of which is consumed by loading the database and filtering out the structures to use for the search. We could estimate from this sample that the program is able to perform an average of 30 full automatic Rietveld refinements per second, per thread.

In Fig. 3 ▸, and in all the following figures, the plotting output from the web page has been visually reformatted during the editing of this paper. In Table 1 ▸ we report the results of the quantitative analysis and compare them with the results of the round robin. Even though the automatic Rietveld analysis was performed using a lot of restrictions, the results are satisfactory and the absolute error was within 2%. Looking at the general results of the round robin (Le Meins *et al.*, 2003 ▸), our automatic analysis scored much better than the average participant result. In fact, the standard deviation on the quantitative analysis results for sample 1*h* reported by the organizers of the round robin is larger than our error. In reality we think that all the restrictions actually help the automatic analysis to avoid possible errors arising from inappropriate setting or refinement of some parameters.

The first test was a simple one, mainly used to check the speed, feasibility and accuracy of the analysis. In contrast, the second part of the test was devoted to comparing the FPSM procedure with the classical search–match routines. For this reason we used the datafiles and tests provided by the search–match round robin (Le Meins *et al.*, 2002 ▸). The round robin considered four samples, and we used two procedures, as in the previous test. In the first procedure, no composition restriction was applied, while in the second, we used the possible elemental composition given by the round-robin proposers to restrict the number of phases during the search. The analysis could not be completed for sample 2, because it contained one unknown crystal structure. There was no similar structure in the COD and the correct phase is only reported in the JCPDS-PDF database as a card. The structure is described as a silicon oxide quinuclidine fluoride octadecasil and we have not found it even in the Cambridge Structural Database (Groom *et al.*, 2016 ▸). So we will report the test results on the other three samples: two inorganics and one organic.

**Figure 4:**
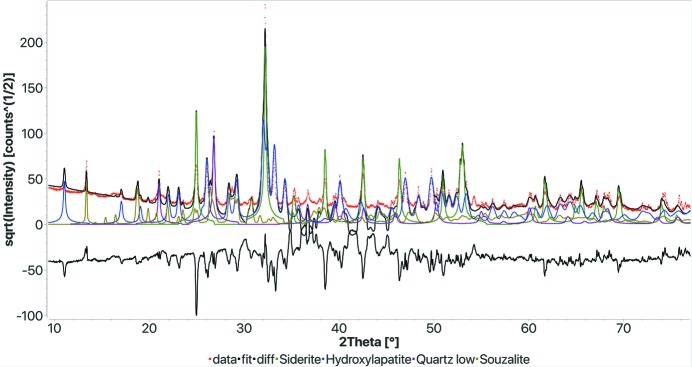
Results of the FPSM on sample 1 of the search–match round robin (Le Meins *et al.*, 2003 ▸). The execution time was 282 s on the Nanoair workstation (2 × 6 cores, 2.93 GHz). *R*_wp_: 0.318.

Sample 1. This sample analysis was performed using the standalone version of the program without compositional restrictions and the web form with restrictions. Both gave the same results, so we report here only the output of the web version. The final results, in line with the round-robin solution, are reported in Fig. 4 ▸ and Table 2 ▸. The main discrepancy in the fit visible in the diagram arises from a small difference between the souzalite structure of the real sample and that available in the database. Souzalite, or the analogue gormanite, has a high variability in composition and structure. In the analysis reported, we limited the phases to no more than five. Otherwise, FPSM will also find and add several other minor phases, all around 1%, that are indeed possible, as the round-robin organizers acknowledge, but we preferred to omit them from the results as there is no information available on them to check for correctness.

**Figure 5:**
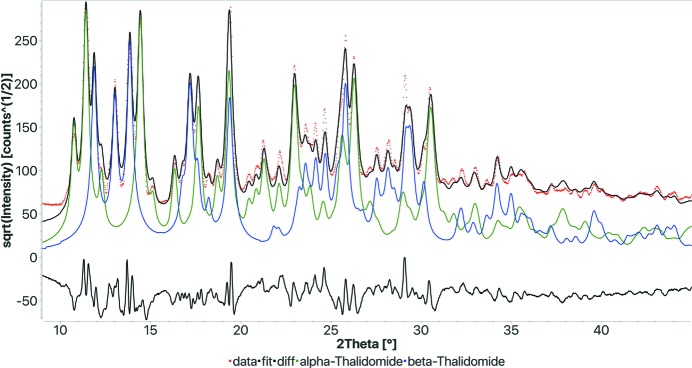
FPSM final fit of the search–match round-robin sample 3. Both α and β polymorphs have been identified as the only present phases. The time for computation was 3307 s. *R*_wp_: 0.153.

Sample 3. This sample is an organic, and according to the notes on the round-robin results it should be composed of about 50:50% of two polymorphs, α- and β-thalidomide. However, the JCPDS-PDF only contains the α polymorph, and consequently identification with only the α form was accepted. In fact, none of the round-robin participants identified the other polymorph. In our case, we did have both the α and the β form in the COD database and FPSM found both correctly (Fig. 5 ▸ and Table 3 ▸). But in this case, even with the compositional restrictions, we could not run the search on the web interface as we have nearly 40 000 organic phases in the database having similar composition. So we only used the standalone version for this test. FPSM took about 3300 s to find the solution on the workstation. The calculation time seems relatively high, but if we consider that we went directly from the raw pattern to the final correct phase quantification, this is reasonable in absolute terms.

**Figure 6:**
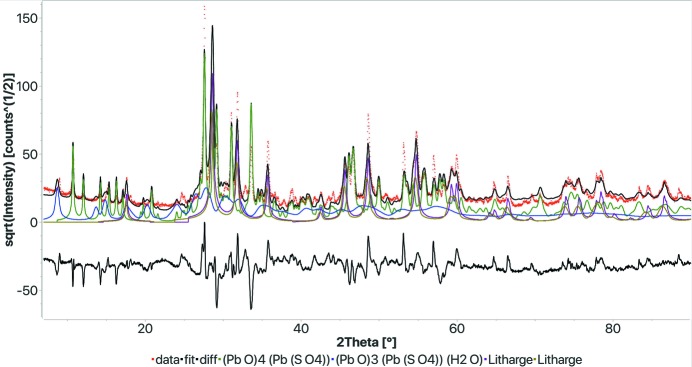
Results of the FPSM analysis on search–match round-robin sample 4. Two PbO phases are present with the same structure, one with large and one with small crystallites to provide a better fitting. Not having the real sample, we do not know if the PbO does have something like a bimodal distribution of grain sizes that may explain the particular peak profile. *R*_wp_: 0.302.

Sample 4 was the easiest one, being composed of only lead-based compounds, but it showed some particularities. Again we report here only the analysis done via the web form using the restricted composition. The unrestricted test gave similar results but in a much longer time. The full analysis was completed in 40 s, giving the correct phases, but in addition several other compounds like sulfur, ice and sulfuric acid were found, showing substantial broadening. These compounds were mainly used by the refinement routine to provide a better fitting of the background modulations. Using a threshold density of 2 g cm^−3^, these spurious phases were eliminated, and the total computation time dropped down to 19 s. The results for the last case are reported in Fig. 6 ▸ and Table 4 ▸. FPSM did not find the massicot phase that the round-robin organizers reported as present. Indeed, many participants did not find it either. So we investigated the presence of massicot further by performing a manual Rietveld refinement with the found phases and the massicot crystal structure. The massicot quantity was always refined close to zero. We notice that all the peaks assigned to the massicot are also peaks of the other phases, apart from a medium-strong peak at 63° 2θ that is in fact not present in the experimental pattern. The organizers did not use the entire range at their disposal but only up to 62° 2θ from their plot. We can safely conclude that the massicot not identified by FPSM is indeed not present in the sample. This example shows an advantage of the present procedure, because by using pattern fitting it may better evaluate the effective presence of a phase by considering the real contribution of the others.

**Figure 7:**
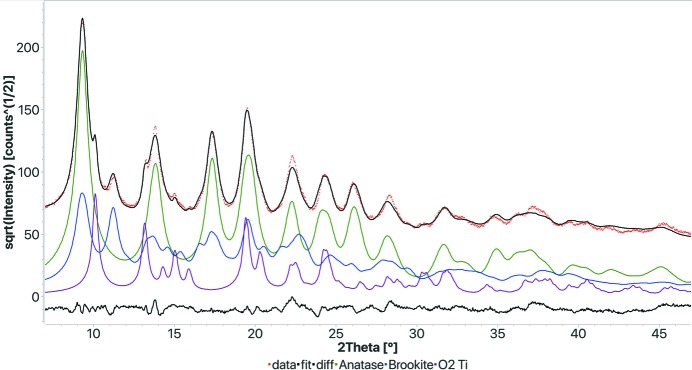
Results of the FPSM analysis for a nanocrystalline titanium oxide sample. All three polymorphs have been found. Pattern collected with an INEL CPS 120° detector and Ag source. *R*_wp_: 0.054.

In addition to the standard and round-robin tests we have analysed other samples, and we will report here a couple of extreme cases. We anticipated that the FPSM procedure may be ideally suited for nanomaterials, where the substantial broadening and overlap make the identification of the peaks and their positions more difficult. One example (TiO_2_ rutile nanoparticles), where we used an early version of FPSM, has already been reported in a paper dealing with TEM diffraction ring analysis (Boullay *et al.*, 2014 ▸). We used another TiO_2_ sample prepared in different conditions (Ceccato *et al.*, 2003 ▸), containing more than one polymorph. The sample has been measured in a capillary using silver radiation and an INEL (now Thermo Fisher Scientific) CPS 120° detector, to reach a large range in *Q* space, the data originally being intended for a pair distribution function analysis. For both the traditional search–match and FPSM we had to impose restrictions using the known chemical elements. It was easy to identify the anatase phase by both procedures (nearly 67 wt% of the sample), while the rutile and brookite phases were ranked very low by the traditional search–match. The results from the FPSM method are reported in Fig. 7 ▸ and Table 5 ▸. All three polymorphs have been correctly identified, and the quantification is not far from the best fit obtained when a manual Rietveld refinement of the crystal structures was conducted with an anisotropic model for the size–strain broadening and the intensity data in the full *Q* range (up to 16 Å^−1^). FPSM on the workstation took 20 s to obtain the final result.

**Figure 8:**
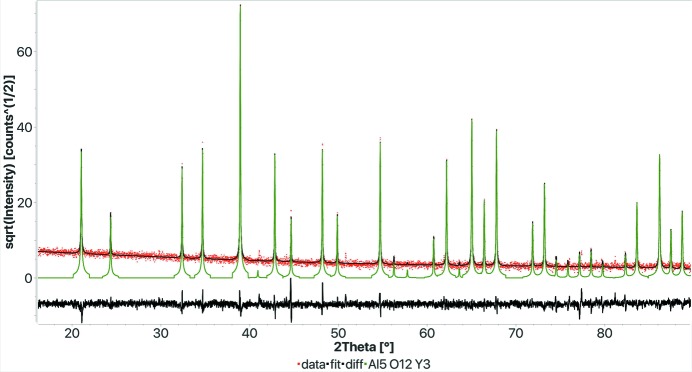
Results of the FPSM analysis for a highly crystallized garnet sample. The total filtering and search time was 1 s when restricting the composition to only phases with Al, Y, Ca and O atoms. Pattern collected with an INEL CPS 590 detector, Co *K*α_1_-only source. In the FPSM web page, we selected ‘Synchrotron’ for instrumental broadening and ‘high’ for crystallization. *R*_wp_: 0.269.

Since the FPSM procedure has been demonstrated to work for a nanocrystalline sample, we wondered how it would perform when we have a highly crystallized sample measured on a high-resolution instrument. In such cases, the peaks become similar to delta functions and the shape of the profile is less significant than the precise reflection positions. Small differences in cell parameters or instrument misalignments may be critical for the procedure presented here. But surprisingly, in all the tests we have done we concluded that there are no drawbacks with sharp peaks. The full-profile search–match is much quicker than in the normal or nanocrystalline cases, and the identification is much easier. As an example, we present a test made on an yttrium aluminium garnet sample for which the possible atoms were Al, Y, Ca and maybe O. The pattern was collected with Co *K*α_1_ radiation (using a Johansson Ge111 crystal monochromator), a capillary and this time an INEL 500 mm radius CPS 590 detector to obtain reasonable intensities in a relatively short time (90 min) but very sharp reflections. Fig. 8 ▸ and Table 6 ▸ report the final results obtained through the web interface. As options we selected ‘high’ for the crystallization of the sample and ‘Synchrotron’ for the instrumental broadening. The entire search and quantification was completed in 1 s using the list of atoms for the composition restriction.

**Figure 9:**
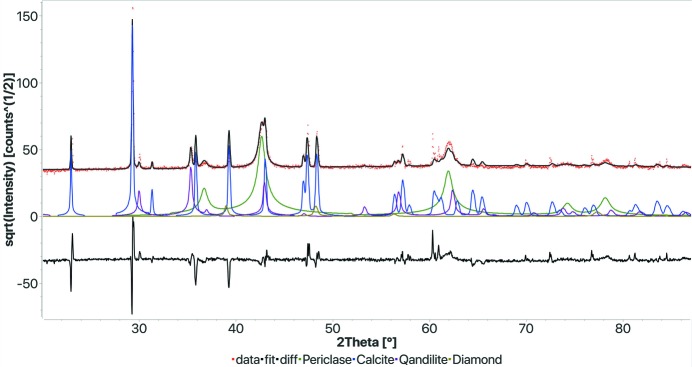
Results of the FPSM analysis for the sample containing nanocrystalline periclase and large grains of calcite and magnetite. The magnetite is less than 3 wt% and not identified owing to the poor statistics on its intensities in addition to the small amount of content. The magnetite peaks are better fitted by the similar qandilite structure. Also, a spurious diamond phase was erroneously found. *R*_wp_: 0.151.

We have tested the method on a few more examples to check its limits. In the first case, we wanted to verify what happens when the sample contains some phases with large grains. In such cases the intensities do not match the theoretical values, and this should affect the results. We used a sample containing a nano-grained periclase, large grains of calcite (high content) and large grains of magnetite (minor content). The sample was measured with monochromated Cu radiation with a fixed incident angle and a 120° CPS detector. No spinning or movements were applied, to emphasize the grain statistics problem. The results of the search–match using FPSM are shown in Fig. 9 ▸ and Table 7 ▸. Calcite, albeit not perfectly matching the reflection intensities, was identified first, followed by periclase. The minor magnetite phase, having large deviations from the intensities of a perfect randomly oriented powder pattern, was not identified, no matter which options we selected in the algorithm. Instead, the qandilite phase was identified, and we found it has a similar structure to the magnetite, the only difference being possible substitution in the Fe^3+^ and Fe^2+^ sites with other atomic species like Mg^2+^, Ti^3+^ or Al^3+^. The diamond phase, which was wrongly found by the algorithm, contributes only to lowering the *R*_wp_ by better fitting of the background and some strong reflections of calcite and magnetite with high graininess. The final quantification may be wrong owing to the carbon phase and graininess, but if we ignore the carbon phase and take qandilite as a replacement for magnetite the result can be considered a partial success. We compared this with the results obtained using the traditional search–match program *QUALX* (Altomare *et al.*, 2015 ▸), also using the COD database. With *QUALX*, after a first automatic peak search, we had to manually remove and add reflections to get a reliable selection. The high background, the noise, and the presence of small, sharp and broad peaks did not permit a satisfactory automatic peak identification. The elemental composition was used to restrict the search as for FPSM. *QUALX* was able to find all the phases correctly, as the intensity problems were not of concern when using software that checks only the peak positions, but except for calcite, which occupied from the top to the seventh position among all candidates in a list of identified phases, the next two structures found were qandilite and finally MgO. The correct phase, magnetite, appeared only in 17th position. We conclude that in this case FPSM gave more or less the same results as the traditional search–match, even in the presence of strong intensity variations. But the traditional search–match required more assistance and human judgement to first identify the correct peak positions and subsequently select the correct phases from the large ranking list. Its computational part was much quicker, but the entire process including human action took much longer.

**Figure 10:**
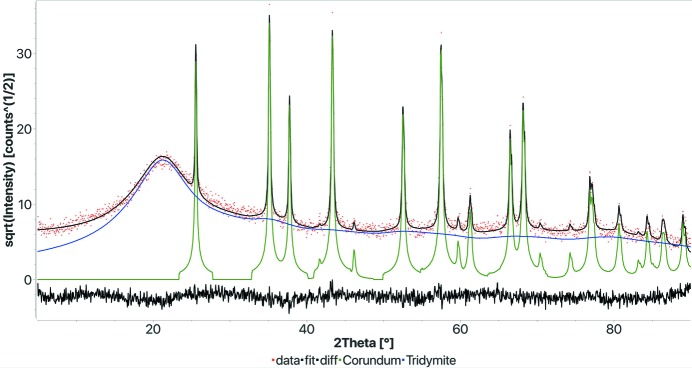
Results of the amorphous FPSM analysis test. The pattern was collected with Cu radiation on a sample containing 50 wt% of pure silica glass and 50 wt% of crystalline corundum. *R*_wp_: 0.138.

Finally we tested the Rietveld search–match on a sample with 50 wt% pure silica glass, the rest of the volume being crystalline corundum. The powder pattern exhibits a large bump between 15 and 25° 2θ, and the original data are available as one of the examples provided with the *MAUD* program. In the first trial we ran FPSM with the default option (50% Rietveld and 50% DDM) to see if it would be able to identify the corundum and not be biased by the amorphous background. To our surprise there was no problem in identifying the corundum as the first phase in the ranking, but FPSM also partly refined the amorphous halo using an SiO_2_ nanocrystalline model and nothing else was found. Indeed, the fit of the amorphous bump was not good. We ran FPSM again using a weight for DDM ten times larger than that for the Rietveld refinement. Again (Fig. 10 ▸ and Table 8 ▸) FPSM immediately and easily identified the corundum, but found a much better fit for the amorphous phase using a tridymite nanocrystalline model. In fact, Le Bail (1995 ▸) has shown how a nanocrystalline model can be used to refine the silica glass structure using a Rietveld-like approach. From the quantitative point of view the result is even more surprising as it gives us the correct amount in weight with an absolute error smaller than 1%, even though tridymite is not the correct structure to use in this case (Lutterotti *et al.*, 1998 ▸).

## 5. Conclusions   

We have described a new method to perform a search–match based on full-profile fitting and the Rietveld/DDM algorithms. It works quite well for highly crystallized samples, but the method looks promising also for nanosized particles and nanomaterials. In the latter case it is generally more difficult to determine unambiguously the peak positions required by the traditional search–match routines. More testing is necessary to determine the algorithm’s efficiency with nanocrystalline systems of greater complexity. The method relies heavily on brute force in computation and the availability of databases of crystal structures. In the present work we used the COD database, but potentially any other structure database can be used.

The main drawback of the method, at present, is that the computation time is much longer than usual search–match programs, unless some compositional restriction can be used. But considering that the method provides a restricted range of phases and a Rietveld quantification through pattern refinement, the running time should be compared with the total time needed to complete not only a search–match but also the further phase selection and a Rietveld pre-quantification. No human intervention is required during the entire process, and therefore the time taken should not be considered problematic. We have tested the method only on a single desktop computer, but for heavy use it would not be difficult to port the system over a cluster to improve its performance and its capabilities to detect minor phases and to solve more complex nanocrystalline problems.

The method is not always able to find minor phases, especially if the background, noise or both are high and the peaks quite broad or not well defined. Removing the background before running the method may partially solve the problem. With the aim of overcoming this problem, we have implemented, alongside the Rietveld refinement, the DDM method (Solovyov, 2004 ▸), which is less dependent on a good background fit. We also found it useful that the DDM method can emphasize the importance of missing peaks that normally contribute marginally to the global fitting and in consequence are generally ignored by the search–match method. This helps to improve the ability to find minor phases. The method is also quite sensitive to the quality of the pattern used, in particular the noise and the background, as they affect the Rietveld refinement. On the other hand, the instrument alignment, affecting the peak positions, and the peak overlap/broadening are less of a concern.

The last major problem we found arises from the completeness of the database used. If the JCPDS-PDF database is considered quite complete, at least for the inorganic case, including both the classical experimental reflections and the ones calculated from the crystal structure databases, the same cannot be said for other crystal structure databases, especially the free ones like the COD. Using a more complete database like the ICSD for inorganics or the CSD for organics may well improve its performance, but it has not been possible for us to test them up to now.

Further improvements could be achieved by integrating a pre-search using a traditional position-based search–match to reduce the initial number of phases. We are also planning to add the ability to deal with preferred orientations as they affect the intensities. Another area to improve is the clay minerals field, for which the incorporation of a smart modelling of the planar defects, like the single-layer model (Ufer *et al.*, 2004 ▸; Lutterotti *et al.*, 2010 ▸), may be helpful. In the future, as the speed of computers grows every day, and crystallographic databases are becoming more complete, these initial concerns should no longer be a problem.

## Supplementary Material

Experimental datafile in CIF format used to test FPSM, results in Figure 7. DOI: 10.1107/S160057671900342X/nb5231sup1.txt

Experimental datafile in CIF format used to test FPSM, results in Figure 8. DOI: 10.1107/S160057671900342X/nb5231sup2.txt

Experimental datafile in CIF format used to test FPSM, results in Figure 9. DOI: 10.1107/S160057671900342X/nb5231sup3.txt

## Figures and Tables

**Table 1:** Results of the FPSM analysis on CPD sample 1*h* from the quantitative analysis round robin (R-R) (Le Meins *et al.*, 2003 ▸), compared with the R-R results Column 4 contains the mean value from all participants in the round robin. In column 5, the mean standard deviation of the analyses performed by all participants using the CPD-supplied data is reported for comparison. In columns 6 and 7, the minimum and maximum values obtained by the participants is shown.

Phase	Wt%	FPSM	Mean R-R	Std. Dev.	Min	Max
Corundum	35.12	37.23	35.96	2.71	30.30	43.83
Fluorite	34.69	33.94	35.21	2.40	30.33	40.30
Zincite	30.19	28.83	28.93	2.56	16.49	33.81

**Table 2:** Results of the FPSM analysis on sample 1 of the search–match round robin (Le Meins *et al.*, 2003 ▸) (see Fig. 4 ▸)

Phase COD ID	Name	Vol.%	Wt%	Crystallites (Å)	Microstrain
9000098	Siderite	39.5	46.0	770.5	0.0009
9002213	Hydroxylapatite	34.9	32.6	1000	0.0004
1011172	Quartz low	13.1	10.2	1000	0.0004
9005624	Souzalite	12.5	11.2	1000	0.0004

**Table 3:** Results of the FPSM analysis on sample 3 of the search–match round robin (see Fig. 5 ▸)

Phase COD ID	Name	Vol.%	Wt%	Crystallites (Å)	Microstrain
1513335	α-Thalidomide	52.3	53.3	444	0.00275
1513336	β-Thalidomide	47.7	46.7	421	0.00104

**Table 4:** Results of the FPSM analysis on sample 4 of the search–match round robin (see Fig. 6 ▸)

Phase COD ID	Name	Vol.%	Wt%	Crystallites (Å)	Microstrain
9012702	Litharge	7.7	8.5	2669	0.004
1513337	(Pb O)4 (Pb (S O4))	45.4	43.9	1259	0.0015
1513337	(Pb O)3 (Pb (S O4)) (H2 O)	17.1	14.4	450	0.00085
9012697	Litharge	29.8	33.2	640	0.00087

**Table 5:** Results of the FPSM analysis on the nanocrystalline titanium oxide sample (see Fig. 7 ▸)

Phase COD ID	Name	Vol.%	Wt%	Crystallites (Å)	Microstrain
1010942	Anatase	65.7	64.3	64.8	0.00356
9004137	Brookite	26.4	27.6	57.9	0.00123
4102355	Rutile	7.9	8.1	152.7	0.00018

**Table 6:** Results of the FPSM analysis on the garnet sample (see Fig. 8 ▸)

Phase COD ID	Name	Vol.%	Wt%	Crystallites (Å)	Microstrain
4312143	Al5 O12 Y3	100	100	3930	0.00026

**Table 7:** Results of the FPSM analysis on the sample with both nanocrystalline periclase and other well crystallized phases (see Fig. 9 ▸)

Phase COD ID	Name	Vol.%	Wt%	Crystallites (Å)	Microstrain
9000095	Calcite	35.7	31.3	3334	0.00132
9000494	Periclase	35.6	40.7	158	0.0001
1011276	Qandilite	4.4	5.1	625	0.00075
9012235	Diamond	24.3	22.9	853	0.018

**Table 8:** Results of the FPSM analysis on the amorphous sample (see Fig. 10 ▸)

Phase COD ID	Name	Vol.%	Wt%	Crystallites (Å)	Microstrain
5000092	Corundum	36.9	50.8	1099.5	0.0007
9005269	Tridymite	63.1	49.2	38.1	0.1124
